# Providers’ perspectives on clinical case consultation following online training in family-based treatment for adolescent anorexia nervosa

**DOI:** 10.1186/s40337-025-01511-8

**Published:** 2025-12-31

**Authors:** Brittany Matheson, Ainsley Cogburn, Aileen Whyte, Daniel Le Grange, James Lock

**Affiliations:** 1 Department of Psychiatry and Behavioral Sciences, Stanford University School of Medicine, 401 Quarry Road, Stanford, CA 94305 USA; 2 Department of Psychiatry and Behavioral Sciences, University of California, San Francisco, CA USA; 3 Department of Psychiatry and Behavioral Neuroscience, The University of Chicago, (Emeritus), Chicago, IL USA

**Keywords:** Clinical case consultation, Provider perspectives, Anorexia nervosa, Evidence-based treatments

## Abstract

**Background:**

Online training programs offer accessible, cost-effective solutions to disseminate evidence-based interventions. Yet, online training is typically insufficient without additional clinical case consultation (CCC). This is particularly salient in adolescent eating disorders treatment, where clinical demand far outstrips capacities of providers trained in evidence-based treatment approaches. This study seeks to better understand attitudes and barriers to receiving CCC among private practice clinicians treating adolescent eating disorders.

**Methods:**

Licensed private practice clinicians (*n* = 47; 100% female, average age 36 y old; 75% master’s degree; average of 4y experience) across the United States enrolled in a randomized trial offering online training (webinar or e-learning) in family-based treatment (FBT) for anorexia nervosa. Post online training, participants were asked before and after 12 sessions of expert CCC to self-report attitudes and barriers to obtaining CCC.

**Results:**

Prior to CCC, participants rated expert CCC in learning FBT as important/very important (100%). The majority participated in CCC since licensure (82%) and in the last year (68%), rating it valuable or very valuable (77%). Participants predicted that CCC would be valuable (96%) and an important motivation in completing the training study (96%). After CCC, participants viewed CCC as important/very important in learning FBT (94%). Common obstacles included finding a study-eligible patient (44%), scheduling constraints (19%), lost wages (16%), mismatch with consultant (3%), and hesitation to discuss cases (3%).

**Conclusions:**

Clinicians reported favorable perspectives on CCC in complimenting learning FBT via online training. Future studies are needed to determine methods to deliver, assess, and scale CCC to enhance treatment fidelity.

**Supplementary Information:**

The online version contains supplementary material available at 10.1186/s40337-025-01511-8.

## Abstract (plain language summary)

This study seeks to better understand attitudes and barriers to receiving clinical case consultation (CCC) among clinicians treating adolescent eating disorders in private practice settings. Clinicians answered questionnaires before and after receiving CCC as part of a larger randomized clinical trial investigating online training methods for family-based treatment (FBT). Private practice clinicians viewed CCC as valuable and important for learning FBT via online training. Common barriers to receiving CCC included practical issues such as finding an eligible study patient, scheduling conflicts, and lost wages. The findings suggest that integrating CCC with online training may be an important component when disseminating evidence-based treatments via online training formats.

**Supplementary Information:**

The online version contains supplementary material available at 10.1186/s40337-025-01511-8.

## Background

Despite the critical need to provide timely, effective treatments for individuals with psychiatric disorders, there are numerous challenges in disseminating effective psychosocial treatments [1–3]. The research to practice pipeline for evidence-based treatments (EBT) is estimated to be 17–20 years, with approximately only 20–50% of evidence-based interventions integrating into standard clinical care [4–7]. Thus, for patients who access care, only a small percentage receive research-supported interventions.

CCC is widely recognized for its benefits to therapists, including increased self-efficacy and skill acquisition [8] and considered to be a collaborative process aimed at developing science-informed practice [9]. Self-assessment by the supervisee as well as bidirectional feedback between supervisee and consultant is encouraged, and goals are set to establish competency and skill acquisition [10]. The learning in CCC has been likened to Kolb’s learning cycle, which includes experiencing, reflecting, conceptualizing, and experimenting [11]. In addition, group consultation may provide benefits such as peer feedback, observational learning, diverse perspectives on issues, and enhanced social connections [12]. However, despite consensus amongst practitioners on the value of CCC both for improving therapists’ overall skill level as well as maintenance of fidelity to treatment model, there is a clear need for more information on how CCC could help improve patient outcomes [13].

In the field of eating disorders, the treatment gap is quite substantial, with approximately 30 million people suffering from an eating disorder but only 10–30% of those diagnosed receiving any treatment, EBT or otherwise [14, 15]. Furthermore, only 6–35% of providers report using EBT in clinical practice for the treatment of eating disorders [16–18]. Traditional training approaches have yet to close this gap and are often not sustainable. One solution to increase trained providers and decrease access to care barriers is online training approaches in evidence-based treatments. Online training methods offer efficient, cost-effective strategies to provide therapists training in EBT through increased access, lower costs, and standardized training procedures [2, 19–22]. However, data suggests that online training alone is not sufficient to achieve clinical outcomes comparable to results obtained in clinical trials and thus additional post-training clinical case consultation (CCC) is needed [23, 24]. The addition of CCC after training poses unique challenges, including additional direct costs in consultant fees, indirect costs in lost productivity for the trainee, and lack of established metrics around how much CCC is needed to achieve competence in delivering a new intervention [25–28].

Family-based treatment (FBT) is an established EBT for adolescent AN [29–32], with sustained recovery rates 3–4 years post-treatment around 40% [33–35]. AN is a psychiatric disorder with a typical onset during adolescence and an incidence rate of around 1% [36–40]. Notably, AN has a high mortality rate, can result in significant medical complications, and is costly to treat [41–44]. Thus, it is imperative to develop substantial, implementable training models that can equip providers with the necessary background and training to treat this illness in clinical practice.

This study reports on attitudes and barriers to receiving CCC among private practice clinicians after completing online training and CCC in an evidence-based treatment (family-based treatment; FBT) for adolescents with anorexia nervosa (AN). The data analyzed was collected as part of a randomized controlled trial comparing the efficacy of two online training modalities for FBT for AN: a webinar training lecture series and an interactive on demand e-training program. The webinar series consisted of 1-hour weekly lectures while the e-training program consisted of 10 self-paced lectures with a focus on key FBT interventions (e.g., agnosticism, externalization). An in-depth description of the study procedures was previously published [45].

## Methods

All participants provided informed consent in accordance with the approved IRB protocol (e-protocol #69550). To be eligible for enrollment, participants were required to have US licensure ((Masters, Ph.D., PsyD, MD (psychiatrist)), treat adolescents with anorexia nervosa in a private practice setting and have no prior training in FBT. Regardless of randomized training conditions (webinar lecture or e-training), therapist participants were asked to complete an 8-item consultation questionnaire after training to assess self-reported attitudes and barriers to obtaining CCC (Online Appendix A). Participants then started group CCC with expert consultants in FBT. In order to join the group CCC, participants had to identify an adolescent patient with a diagnosis of AN who needed at least 5 pounds of weight gain and provide FBT for that patient in their private practice setting. This case would then be discussed in the CCC groups. Consultation groups ranged in size from 1 to 4 participants. Participants were asked to complete at least 4 sessions of CCC, though could partake in up to 12 sessions total. After the fourth session of CCC, participants were re-assessed and completed the 8-item consultation questionnaire again about their experience.

## Results

Participants (*n* = 47; 100% female; M + SD: 36y + 6.7) were licensed private practice clinicians across the United States (Table 1). Twenty-two participants completed the pre-CCC assessment survey and thirty-two participants completed the post-CCC assessment survey; only seven participants completed the assessment at both time points. On average, participants reported 3.95 (SD: 4.0) years of private practice experience and most participants were master’s level clinicians.

**Table 1:** Participant demographics (*N* = 47)

	M	(SD)
Age	36	6.71
Years in private practice	3.95	4.04
	**n**	**(%)**
Sex-female	47	100
Education		
Masters	35	(74.46)
PhD	6	(12.77)
PsyD	5	(10.64)
MD	1	(2.13)
Race		
White	44	(93.62)
African American/Black	0	(0.00)
Asian	0	(0.00)
Native Hawaiian/ Pacific Islander	0	(0.00)
Multiracial	3	(6.38)
Ethnicity– non-Hispanic/Latinx	43	(95.55)

### Before CCC: attitudes and potential barriers

Prior to starting CCC, participants rated expert CCC in learning FBT to be important or very important (Table 2). The majority reported previous participation in CCC since licensure and in the last year and found it valuable/very valuable. All but one participant predicted the FBT CCC would be valuable and an important reason influencing study participation.

**Table 2:** Clinical case consultation questionnaire

	EOT (*n* = 22)		FU (*n* = 32)	
	*n*	(%)	*n*	(%)
Importance of expert consultation				
Moderately important	–	–	1	3.1
Important	3	13.6	7	21.9
Very Important	19	86.4	23	71.9
No Response	–	–	1	–
Participation in supervision since licensure				
Yes	18	81.8	29	90.6
No	4	18.2	3	9.4
Value of past case consultation				
Slightly valuable	–	–	2	6.3
Moderately valuable	–	–	1	3.1
Valuable	5	22.7	9	28.1
Very valuable	12	54.5	16	50
No response	5	22.7	–	–
Participated in case consultations within the past year				
Yes	15	68.2	27	84.4
No	3	13.6	2	6.3
No response	4	18.2	3	–
Obstacles to obtaining case consultation				
Finding a patient that meets criteria	16	72.7	14	43.8
Scheduling constraints	8	36.4	6	18.8
Mismatch between you and consultant	2	9.1	1	3.1
Hesitation to discuss cases in front of others	1	4.5	5	15.6
Lost wages	1	4.5	–	–
No obstacles anticipated	5	22.7	13	40.6
FBT consultation is valuable				
Strongly Agree	15	68.2	24	75
Agree	6	27.3	7	21.9
Undecided	1	4.5	1	3.1
I look forward to the FBT consultation				
Strongly Agree	14	63.8	20	62.5
Agree	7	31.8	11	34.4
Undecided	–	–	1	3.1
Strongly disagree	1	4.5	–	–
The FBT consultation was an important part of why I signed up for this research study.				
Strongly Agree	10	45.5	20	62.5
Agree	11	50	5	15.6
Undecided	–	–	6	18.8
Disagree	–	–	1	3.1
No response	1	4.5	–	–
Barriers to finding a patient for supervision?				
Yes	15	68.2	15	46.9
Patient weight	6	27.3	8	25
Patient diagnosis	4	18.2	4	12.5
Patient age	5	22.7	4	12.5
Not seeing AN patients regularly	3	13.6	2	6.3
Pt/family not in agreement to receive FBT	5	22.7	7	21.9
Patient comorbidity	–	–	2	6.3
Concerns about reporting patient weight	–	–	2	6.3
Other	5	22.7	1	
No	7	31.8	17	53.1

When asked to reflect on potential obstacles with attending CCC, finding an eligible patient for the study was the most cited concern, followed by scheduling constraints, mismatch between provider and consultant, hesitation to discuss cases in group format, and lost wages. No participants endorsed the option “consultation not a good use of time” or provided other potential obstacles that could interfere with participation in CCC.

Most respondents reported encountering barriers to finding a patient for CCC, with the most common reasons being patient characteristics required for the research study (adolescent with a diagnosis of AN with at least 5 pounds weight gain needed), patient age outside 12–18 years old, patient or family not in agreement about receiving FBT, and not seeing patients with AN regularly in practice. Other reasons noted included potential patients who met study eligibility criteria being readmitted to the hospital or referrals from higher levels of care for FBT in which patients were already weight restored. Participants were also asked to respond with free text to the prompt “any other barriers we did not ask about”; two participants responded with one provider noting “*in between transitions with jobs currently*” and the other provider stating, “*availability of an FBT referral*”. No other potential barriers were identified.

### After CCC: attitudes and barriers

After completing CCC in the study, all but two participants viewed expert CCC in learning FBT as being important or very important. When reflecting on obstacles, finding a patient that met study eligibility criteria was the most common obstacle, followed by scheduling constraints, lost wages, mismatch between provider and consultant, and hesitation to discuss cases in group format. A sizeable portion of private practice providers surveyed said there were no obstacles encountered. All but one respondent indicated that FBT CCC was valuable and they looked forward to the time. Nearly half the sample endorsed barriers to finding a patient for CCC, citing patient weight, patient/family not in agreement to receive FBT, age, diagnosis, comorbidity, and concerns about reporting weight. One participant commented about their experience: “*parents struggle to commit to the full treatment process. Many times*,* fathers would not make time to attend and also scheduling on the families [sic] side was difficult to meet with all siblings present.”*

Nine additional responses were collected by free text to the question “any other barriers not already asked”. Responses highlighted challenges posed by communication with study team members and aligning treatment with CCC, consistency of patients in care, and unpredictability of referrals in private practice settings.

## Discussion

This study was designed to explore attitudes and barriers to completing CCC among clinicians in private practice learning an EBT for adolescents with AN. Overall, clinicians anticipated CCC to be useful and valuable in learning FBT and continued to report high levels of satisfaction post-CCC. While some participants noted concerns related to scheduling constraints and lost wages in attending CCC, the majority of respondents did not endorse these barriers. The eligibility of patients that qualified based on study criteria and were interested in FBT appeared to be the most significant challenge for proceeding with CCC for the clinicians.

Clinician attitudes toward EBP and training are an important challenge to implementing effective treatments in community settings. Attitudes toward supervision in clinical practice also vary by provider and may change with experience. Younger clinicians are often more open to continued learning whereas more experienced clinicians often rely on previous learning or experience [46]. Supervision is often part of training programs, but much is not known about the supervisee’s attitudes towards supervision. In clinical practice, little is known about expectations of clinicians related to consultation, with systematic reviews documenting a lag in supervision research compared to EBP outcomes research [47].

Strengths of this study include two assessment points, before and after CCC, to compare perceived versus experienced attitudes and barriers to CCC participation. Other strengths include a nationwide sample and limited restrictions on type of mental health professional discipline (other than licensed and in good standing) to increase generalizability. There are several limitations to note, including different participants responding at different time points and thus pre/post individual participant analyses were not feasible. Additionally, only 47 participants answered this questionnaire, and thus the attitudes and barriers of folks who participated in the online training and CCC portions of the trial but did not complete this assessment are unknown. It is possible that these individuals experienced similar or unique challenges in attending CCC that cannot be extrapolated based on the available data obtained. Thus, the missing data from participants who did not complete the surveys and/or discontinued participation in the training and CCC portions of the trial limits conclusions and generalizability, and introduces unknown response biases. Further, the racial and ethnic diversity in this sample was limited and findings regarding attitudes and barriers to CCC may not be generalizable or representative of all clinicians treating eating disorders in all private practice settings. The difficulty that some clinician participants had in securing a patient for CCC is a challenge unique to the design of the current study and may not represent of a challenge in future studies with less strict patient inclusion criteria. It would also have been helpful if further additional qualitative data could have been collected to better understand what therapists valued about CCC and how they might suggest improving it.

## Conclusions

In summary, private practice clinicians in this study reported favorable perspectives on CCC in complementing learning FBT via online training. Future studies should examine whether clinicians in health services or employed in treatment organizations share similar attitudes toward CCC. Further, it is unclear how much CCC post online training would be valued. In the current study, most of those who started CCC completed all possible sessions offered (12 total), but whether this would be the case in other practices or in a larger sample is unknown. Additionally, research is needed to better understand the impact of CCC on patient outcomes and therapist fidelity to evidence-based treatments, such as FBT. Future studies are needed to examine how best to deliver, assess, and scale CCC as part of overall training and fidelity to EBP.

## Supplementary Information

Below is the link to the electronic supplementary material.

Supplementary Material 1.

## Data Availability

Data from the current study are available upon reasonable request.
