# Supplementary material for: Providers’ perspectives on clinical case consultation following online training in family-based treatment for adolescent anorexia nervosa
Source: J Eat Disord. 2025 Dec 31;14:39. doi: 10.1186/s40337-025-01511-8 (PMC12857007; doi:10.1186/s40337-025-01511-8)
Supplement: Supplementary file 1 — Supplementary Material 1. [file 40337_2025_1511_MOESM1_ESM.docx]

Appendix A. Copy of consultation questionnaire

| **1** | **How important is expert case consultation in learning FBT?** | | | | | | | |
| --- | --- | --- | --- | --- | --- | --- | --- | --- |
|  | Not important Slightly important Moderately important Important Very Important | | | | | | | |
| **2** | **Have you participated in case consultation or supervision since you were licensed?** | | | | | | | |
|  | Yes No | | | | | | | |
| **3** | **What obstacles have you had or do you foresee in participating in the case consultation portion of this study? Please choose all that apply.** | | | | | | | |
|  | Finding a patient that meets criteria | Scheduling constraints | Mismatch between you and consultant | Hesitation to discuss clinical cases in front of others | Lost wages | Consultation is not a good use of time | Other – please explain | Do not anticipate any obstacles |
| **4** | **The FBT consultation was or will be valuable.** | | | | | | | |
|  | Strongly disagree Disagree Undecided Agree Strongly Agree | | | | | | | |
| **5** | **I look(ed) forward to the FBT consultation.** | | | | | | | |
|  | Strongly disagree Disagree Undecided Agree Strongly Agree | | | | | | | |
| **6** | **The FBT consultation was an important part of why I signed up for this research study.** | | | | | | | |
|  | Strongly disagree Disagree Undecided Agree Strongly Agree | | | | | | | |
| **7a.** | **Are you encountering or did you encounter any barriers to finding a patient for supervision?** | | | | | | | |
|  | Yes No | | | | | | | |
| **7b.** | **If yes, what were they? Please check all that apply.** | | | | | | | |
|  | Patient weight | Patient diagnosis | Patient age | Patient comorbidity | Not seeing patients with Anorexia Nervosa regularly | Concerns about reporting patient weight | Patient/family not in agreement to receive FBT | Other: __________ |
| **8** | **Any other barriers that we did not include regarding your participation in the FBT consultation portion of this study?** | | | | | | | |
|  | _________. | | | | | | | |
